# HGF/Met-Signaling Contributes to Immune Regulation by Modulating Tolerogenic and Motogenic Properties of Dendritic Cells

**DOI:** 10.3390/biomedicines3010138

**Published:** 2015-03-03

**Authors:** Jessica Hübel, Thomas Hieronymus

**Affiliations:** 1 Department of Cell Biology, Institute for Biomedical Engineering, Medical Faculty, RWTH Aachen University, Pauwelsstrasse 30, 52074 Aachen, Germany; E-Mail: jessica.huebel@rwth-aachen.de; 2 Helmholtz Institute for Biomedical Engineering, RWTH Aachen University, Pauwelsstrasse 20, 52074 Aachen, Germany

**Keywords:** Met, HGF, dendritic cells, Langerhans cells, immune tolerance, immune-based therapies, migration, MMPs, epithelial-mesenchymal transition

## Abstract

Hepatocyte growth factor (HGF)-signaling via Met can induce mitogenic, morphogenic, and motogenic activity in various cell types. Met expression in the immune system is limited to cells with antigen-presenting capacities, including dendritic cells (DCs). Thus, it appears highly conceivable that Met-signaling impacts on adaptive immune responses. However, the mechanisms by which HGF imparts its effects on immunological responses are not yet fully understood. DCs possess unique functionalities that are critically involved in controlling both tolerance and immunity. HGF conveys immunoregulatory functions, which strongly correlate with that of DCs orchestrating the apt immune response in inflammation. Therefore, this review focuses on the current knowledge of Met-signaling in DCs with specific emphasis on the morphogenic and motogenic activities. HGF has been identified to play a role in peripheral immune tolerance by directing DC differentiation towards a tolerogenic phenotype. In skin immunity, Met-signaling was shown to drive mobilization of DCs by regulating matrix metalloproteinase activities. This is strikingly reminiscent of the role of Met for regulating a cell fate program during embryonic development, wound healing, and in tumor invasion known as epithelial-mesenchymal transition (EMT). Thus, the concept emerges that an EMT program is executed by Met-signaling in DCs, which will be also discussed.

## 1. Introduction on the Immunoregulatory Function of HGF/Met

Met, a high affinity transmembrane tyrosine kinase receptor for hepatocyte growth factor (HGF) was originally identified as proto-oncogene [1]. HGF was first described as a mitogenic factor for hepatocytes [2] and independently as scatter factor (SF), a motility factor for epithelial cells [3,4] that express Met in various organs during embryonic mouse development [5]. It was demonstrated that conventional null mutations of *Hgf* or *Met* in mice are embryonically lethal, emphasizing the role of HGF/Met-signaling for a proper development [6,7,8]. Later during lifetime, HGF/Met has a vital role in liver [9,10] and pancreatic β-cell [11] regeneration, and in wound healing [12,13,14]. Met-signaling leads to the transient conversion of cells from an epithelial to a mesenchymal phenotype (epithelial-to-mesenchymal transition, EMT) followed by well-controlled cell migration [15,16]. Similarly, aberrant Met-signaling contributes to tumorigenesis and induces invasive growth and metastasis [17,18].

The tissue regenerative function of HGF/Met correlates directly with immune regulation and inflammation [19]. For example, HGF is produced amongst others by platelets [2], neutrophils, and mast cells [20] and acts on various cell types involved in regenerative processes. A role of HGF/Met in autoimmune diseases has been also suggested [21,22]. Consequently, HGF/Met has been considered as a potential target for immune-based clinical therapies [19].

While Met is predominantly expressed by epithelial cells, various hematopoietic cells are in addition able to acquire Met expression. In the hematopoietic compartment and therewith in the immune system, Met expression is limited to hematopoietic progenitor cells and their progenies with antigen-presenting capacities, including monocytes/macrophages, B cells, and dendritic cells (DCs). Met-signaling was found to play a role in monocyte-macrophage development [23] and in regulating B cell homing to the lymph nodes [24]. Likewise, Met expression was shown to regulate splenic DC function [25,26]. Met expression was found on bone marrow (BM)-derived DCs and dermal DCs (dDCs) and Langerhans cells (LCs; the epidermal contingent of DCs), which play a pivotal role in skin immunity [27,28]. Consequently, it appears highly conceivable that HGF-signaling via Met impacts on adaptive immune responses.

## 2. Dendritic Cells Are Key Regulators of the Immune System

DCs are the most professional antigen-presenting cells (APC) of the immune system. They are part of the innate immune system and are distributed in lymphoid and non-lymphoid organs throughout the body where they serve as sentinels of the immune surveillance system [29].

DCs originate from self-renewing hematopoietic stem cells (HSCs) in the BM that develop into progenitor cells. To fulfill their function progenitor cells are obliged to migrate via the peripheral blood towards lymphoid and non-lymphoid tissues where they differentiate into immature DCs [30,31]. Upon encountering foreign pathogens DCs take them up and process them, which includes degradation and peptide/antigen loading on major histocompatibility complex class I or II (MHCI or MHCII) molecules depending on the endogenous or exogenous origin of the pathogen, respectively. MHC and antigen complexes are expressed on the cell surface for recognition by CD4+ (MHCII) and CD8+ (MHCI) T cells [32]. Thereby, DCs are the key initiators and regulators of adaptive immune responses. Interestingly, DCs exhibit the unique property to process exogenous antigens and load them on MHCI through a process referred to as cross-presentation. This represents an important mechanism to act against intracellular pathogens (which are not present in DCs themselves) or tumor cells by induction of a cytotoxic CD8+ T cell response [33,34].

Hence, DCs possess unique functionalities that are critically involved in controlling and maintaining the balance between tolerance and immunity, thus developing an effective immune response and at the same time limit self-damage. This makes DCs attractive targets for immunotherapies, such as vaccines against cancer [35], treatment of graft-*versus*-host disease following transplantations, and in infectious or autoimmune diseases [36].

In this respect it is interesting that HGF mediates immunoregulatory activities, which correlate with that of DCs orchestrating an appropriate adaptive immune response. Moreover, although the mechanisms by which HGF/Met effects immunological functions are not yet fully understood an impact of HGF/Met-signaling on DC functions in splenic DCs [25], as well as on dDCs and LCs was identified [27,28].

## 3. HGF Mediates Development of Tolerogenic Dendritic Cells (DCs)

In peripheral immune tolerance HGF has been identified to play a role by impairment of DC activation characterized by an obstructed antigen presenting capacity [26,37]. In the study by Okunishi *et al.* [26] no detectable interleukin (IL)-10 secretion, a cytokine that suppresses DC function in an autocrine manner [38], was found. Other studies however showed the contrary result, where HGF inhibited immunogenic DC function by stimulating autocrine IL-10 secretion [39,40].

In murine autoimmune models and in human monocyte derived DCs, HGF imparts DCs with suppressive competence resulting in effective induction of regulatory T cells (Tregs) [25,39,41]. Supplementation of differentiating DCs with HGF directed DC development towards a tolerance inducing DC phenotype that increases their capacity of Treg induction. With the enhancement of HGF induced Tregs, other effector T cell subsets, such as T helper (Th)1 and Th17 cells, were concurrently down regulated.

HGF treated DCs were found to be characterized by an increased expression of programmed-death ligand 1 (PD-L1) and IL-27, factors mediating DC-driven Treg generation [41]. In addition to PD-L1 and IL-27, DCs also exhibited HGF-induced increase in glucocorticoid-induced leucine zipper (GILZ) expression [41], which was found to mechanistically impair DC function by interfering with NF-κB-signaling [41,42]. Notably, GILZ expression was shown in previous studies to be a common property of tolerogenic DCs preventing their immunostimulatory activity [43,44]. The interference of HGF on NF-κB-signaling was also shown before to involve activation of Bruton’s tyrosine kinase, a proximal component of the Met-signaling pathway and the PI3K/Akt pathway [39]. These findings indicate that HGF exerts its potent immunoregulatory function by directing DC differentiation towards a tolerogenic phenotype using specific signaling pathways. These findings open the way for drug-mediated interventions on Met-signaling for immune-based therapies.

## 4. Met-Signaling Impacts on DC Migration

### 4.1. Met-Signaling in Monocytes

Monocytes are a leukocyte sub-population with the capacity to differentiate into tissue macrophages and DCs under inflammatory conditions [30]. In recent years evidence accumulated that HGF regulates monocyte and macrophage function including cellular migration. First studies showed that in activated human monocytes under conditions resembling inflammation, Met expression is upregulated [23,45]. Further studies confirmed enhanced Met expression upon monocyte activation by inflammatory signals, which made them accessible for HGF and its functional effects. Stimulation of monocytes with HGF induced increased matrigel invasion and upregulation of proinflammatory cytokines and chemokines, such as IL-4, Il-1β, GM-CSF, and MIP-1β suggesting a proinflammatory role of HGF [46]. Such a role was later confirmed in a murine model of experimental autoimmune encephalomyelitis [47]. It was further reported that inflammatory signals induce activity of cellular pro-HGF convertase that activates the inactive HGF pro-peptide into its active form by proteolytic cleavage and thus increasing monocyte responsiveness to HGF. Furthermore, it was shown that activated monocytes secrete HGF assuming autocrine signaling [48]. Most interestingly, functional migration assays revealed a potent function of HGF to induce directional migration in monocytes [46,48].

### 4.2. Met-Signaling in DC Motility and Migration

HGF-induced migration of monocytes resembles the crucial motile property of DCs for executing their immune function. The motogenic capacities of activated DC are required for emigration form peripheral tissues and migration towards lymphoid organs where they instruct antigen-specific T cells. For this purpose, DC subpopulations in peripheral tissues, such as the skin, constitute a first immunological barrier to the external environment. In skin, dDCs and LCs were shown to express Met, and binding of its ligand HGF effectively activated Met-signaling [27,28]. This augmented DC adhesion to laminin, an extracellular matrix component without affecting APC function [27]. Additionally, skin explant experiments demonstrated that Met activation induces dDC and LC emigration from skin [27,28].

These findings were supported using a conditional Met deficient mouse model where activated skin resident DCs failed to migrate towards the skin-draining lymph nodes despite an activated phenotype [28]. This establishes Met-signaling as a critical determinant of DC/LC detachment from the peripheral tissue and emigration from the skin upon stimulation. Moreover, the data suggest that Met-signaling drives mobilization of LCs and dDCs via matrix metalloproteinases (MMP) activation. It was found that Met is essential for DC migration through the extracellular matrix, which requires MMP activities for matrix degradation. Indeed, both MMP-2 and MMP-9 activity were found to be regulated by Met in DCs [28]. This is in line with previous studies that showed that MMP-2 and MMP-9 were critically involved in DC migration [49,50,51]. Similar to the effect of Met-signaling on DCs, HGF-mediated migration of human corneal epithelial cells [52] and keratinocytes in wound healing was dependent on MMP-9 activity [53]. The concurrence of identical Met-driven mechanisms in different cellular systems such as epithelial cells and DCs point further to the direct correlation of tissue regeneration with inflammatory responses.

## 5. A Met-Driven Program of Epithelial-Mesenchymal Transition in DCs?

HGF-induced DC mobilization and migration upon activation is associated with various phenotypic transformations that includes for example MMP activation and detachment from surrounding tissue. This is strikingly reminiscent of a Met-signaling driven mechanism seen during embryonic development, wound healing, and invasive growth of tumors known as epithelial-mesenchymal transition (EMT). In this process activated immobile epithelial cells convert transiently into a migratory mesenchymal phenotype [15,16]. Thus, the concept emerges that an EMT (or EMT-like) program is executed by Met-signaling in DCs. Similar to epithelial cells, where this program was shown to be Met driven, dDCs and LCs need to disrupt their physical contact to neighboring cells mediated by adherens and tight junctions [54,55]. EMT is characterized by downregulation of components that form adherens and tight junction complexes, including EpCAM, occludins, claudins, zonula occludens (ZO) proteins, and cytokeratins and thereby decomposes cell-to-cell contacts. Simultaneously, cells gain a mesenchymal phenotype, such as expression of N-cadherin, vimentin, integrins, and MMPs and reorganization of the cytoskeleton, which altogether facilitate cell migration. Again, the proteolytic activity of MMPs, stimulated by Met-signaling was shown to facilitate tumor cell dissociation and scattering [54,56]. Another hallmark of EMT is the loss of E-cadherin expression, which is regulated by the zinc finger and E-box binding transcription factors (ZEB) 1 and 2 [55,56,57].

LCs in skin were shown to express E-cadherin and various other epithelial-like junctional proteins, including claudin-1, EpCAM/TROP1, TROP2, ZO-1, occludin, JAM1, and cytokeratins, which allows them to functionally integrate into the keratinocyte layer. Moreover, activation of LC was found to induce expression of the EMT regulators ZEB1 and ZEB2 and downregulation of E-cadherin and EpCAM, accompanied by upregulated expression of N-cadherin [58]. Together with the above described regulation of MMPs in DCs, these findings strongly supports the concept that a Met-signaling initiated EMT-like program is executed in DCs upon activation.

## 6. Conclusions and Perspectives

**Figure 1:**
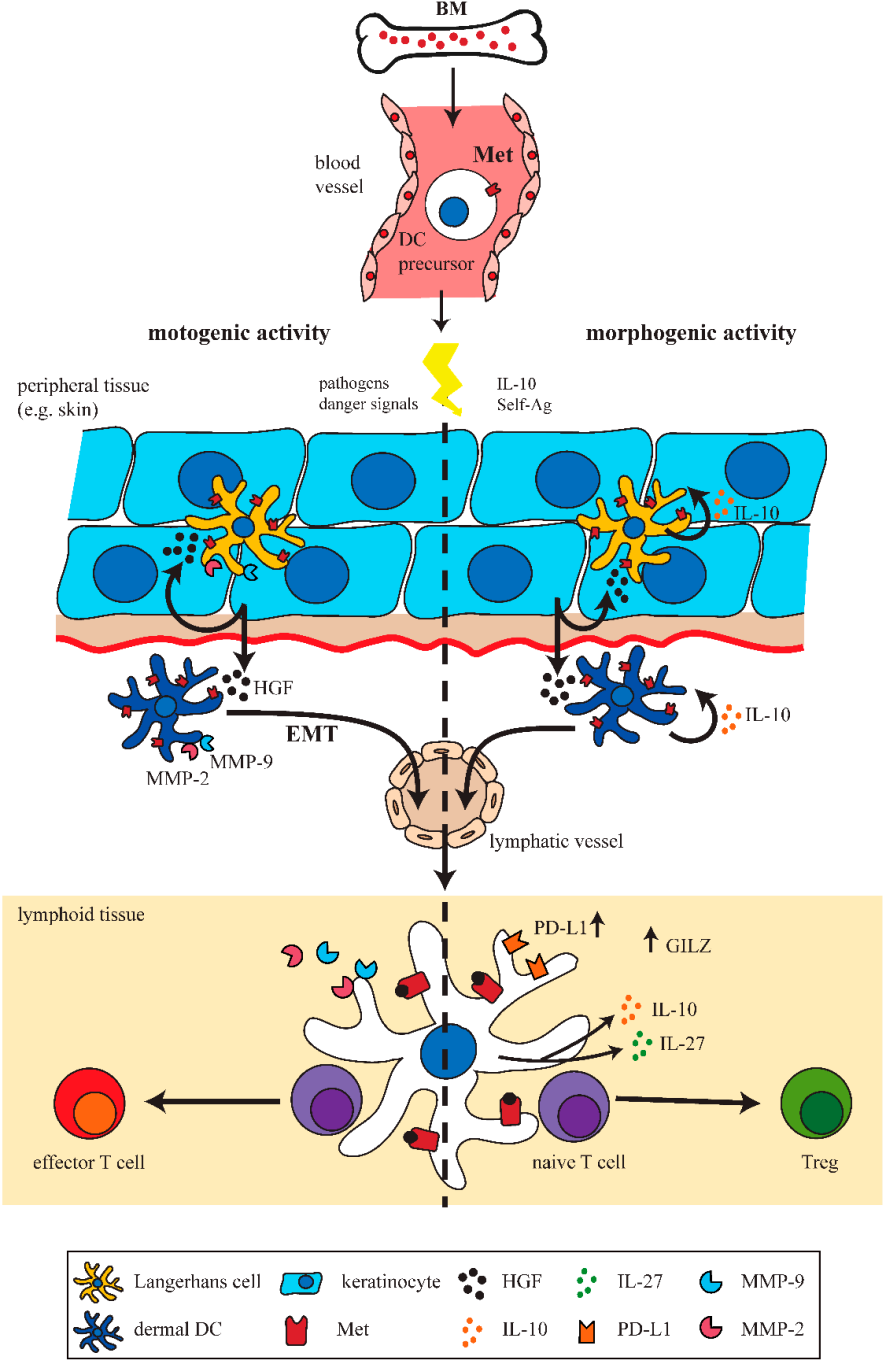
Hepatocyte growth factor (HGF)/Met-signaling in dendritic cells (DCs). Schematic representation of the motogenic and morphogenic activities of Met-signaling on DCs in peripheral and lymphoid tissues. DC precursors originating from hematopoietic stem cells (HSC) in bone marrow (BM) migrate towards peripheral tissues such as the skin. Upon activation DCs/Langerhans cells (LCs) migrate via lymphatics to draining lymph nodes to present antigens to naive T cells. Met-signaling induces LC and DC emigration from skin in an epithelial-mesenchymal transition (EMT)-like process, including matrix metalloproteinases (MMP) activation. HGF-induced morphogenic activities include induction of a tolerogenic phenotype of DCs by IL-10 secretion and upregulated expression of, e.g., glucocorticoid-induced leucine zipper (GILZ) and programmed-death ligand 1 (PD-L1), which finally results in enhanced numbers of Tregs.

Altogether this review summarizes the pivotal role of HGF/Met-signaling in DC mediated immunity (Figure 1). HGF/Met-signaling enables DCs to be directed into two distinct immunomodulatory pathways, either conferring immune tolerance or immunity. In the case of tolerogenic DCs, the influence of HGF has been extensively studied indicating that HGF triggers the IL-10-dependent mechanism of tolerance induction. Furthermore, several proteins restricted to tolerogenic DC were identified as specific HGF/Met-signaling pathway components. Accumulating data indicate that HGF protects against autoimmunity via DC-dependent mechanisms in a number of studies, for example in a murine lupus nephritis model of chronic graft-versus-host disease [59], collagen-induced arthritis [60], experimental autoimmune encephalomyelitis [25,41] and chronic airway inflammation [26].

In contrast, the role of HGF/Met-signaling for DC migration is less well established. Initial studies highlighted the importance of Met-signaling for control of DC motility and migration. First fundaments towards understanding Met-dependent mechanisms in DC migration were established by elucidating MMP regulation by Met-signaling. However, knowledge about intracellular signaling transduction pathways that control DC migration following Met activation is not yet fully exploited. Based on the notion that in wound healing keratinocyte and DC migration are regulated by the same Met-driven mechanisms, it is highly conceivable that similar protein signaling network dynamics exist in DCs and keratinocytes [15,54,61].

Met has been examined in numerous cancer-based studies as a critical factor for tumor invasion and metastasis and thus is highly considered as a drug target for tumor therapies. Accordingly, control of DC function by HGF/Met-signaling as a crux for DC-based immunity represents a potential starting-point for immune therapy against cancer and autoimmune diseases. However, means to target HGF/Met in order to favor immune tolerance might at the same time boost potential tumor cell towards invasive growth and metastasis. The other way round, blocking Met-induced migration of tumor cells in patients might alter their immune function towards development of autoimmune reactions. Nevertheless, this would be a matter of considering the pro and cons, depending on the required therapy and taking into account whether therapies are only temporarily applied.

In summary, the knowledge of intracellular HGF/Met-signaling mechanisms in DCs is still incomplete and needs to be expanded by further research to allow save Met-based therapies in the future. Elucidation of downstream signaling pathways of Met involved in DC migration will also reveal whether a Met-driven EMT program is in fact operating in DCs.
